# Subjective wellbeing of preschool children

**DOI:** 10.3389/fpubh.2023.1156755

**Published:** 2023-04-20

**Authors:** Bianca Núbia Souza Silva, Bianca Gonzalez Martins, Lucas Arrais Campos, João Marôco, Juliana Alvares Duarte Bonini Campos

**Affiliations:** ^1^Department of Morphology and Children's Clinics, São Paulo State University (UNESP), School of Dentistry, Araraquara, São Paulo, Brazil; ^2^Department of Biological Sciences, School of Pharmaceutical Sciences, São Paulo State University (UNESP), Araraquara, São Paulo, Brazil; ^3^Faculty of Medicine and Health Technology, Tampere University, Tampere, Finland; ^4^Department of Ear and Oral Diseases, Tampere University Hospital, Tampere, Finland; ^5^Institute of Dentistry, Faculty of Health Sciences, University of Eastern Finland, Kuopio, Finland; ^6^William James Center for Research (WJCR), University Institute of Psychological, Social, and Life Sciences (ISPA), Lisbon, Portugal; ^7^Flu Pedagogy, Nord University, Bodø, Norway

**Keywords:** subjective wellbeing, child wellbeing, children, preschool, psychometrics

## Abstract

**Objective:**

The aim of the present study was to evaluate the psychometric properties of the *Autoquestionnaire Qualité de Vie Enfant Imagé* (AUQEI) in pre-school children and estimate the influence of demographic characteristics on their subjective wellbeing.

**Methods:**

Construct validity was estimated using confirmatory analysis and the chi-square per degrees of freedom ratio (χ^2^/df), Comparative Fit Index (CFI), Tucker-Lewis Index (TLI), and Root Mean Square Error of Approximation (RMSEA). Reliability was assessed by the ordinal alpha (α) and omega (ω) coefficients and the factorial invariance by the difference in CFI (ΔCFI). Mean scores for each AUQEI item and the general score were calculated.

**Results:**

A total of 443 Preschool children enrolled in public education institutions participated. The original 4-factor AUQEI model showed collinearity between factors and a high correlation between two items. A single factor model was tested, presenting adequate fit to the data (χ^2^/df = 4.47; CFI = 0.98; TLI = 0.98; RMSEA = 0.08; α = 0.98; ω = 0.93; UniCo > 0.95, EVC > 0.85, and MIREAL < 0.30) and strict model invariance (ΔCFI < 0.01). The AUQEI model proved to be valid in relation to the external variables. Most children (76.7%) had positive subjective wellbeing. Higher scores were observed for items concerning recreation, holidays, and birthdays, and lower scores for those referring to hospitalization, medication, medical consultation, and being away from the family. The relationship between the demographic characteristics of the child or his/her mother and subjective wellbeing was not significant (*p* > 0.05).

**Conclusions:**

The assessment of subjective wellbeing with the single-factor AUQEI model provided valid, reliable, and invariant. Thus, being a relevant and interesting instrument to assess wellbeing in young children.

## 1. Introduction

Subjective wellbeing is the self-assessment of life according to different personal criteria, and is based on a conscious complex and multidimensional cognitive judgment (1). In the search for definitions of wellbeing in the literature it is possible to find different ways of evaluating life or emotional experiences that involve feelings, living conditions, experiences, desire satisfaction, and the balance between pain and pleasure (1, 2). According to the literature it is possible to find two different classifications for wellbeing, which are the hedonic and eudaimonic dimensions. Eudaimonic is driven by the cultivation of wellbeing based on long-term emotional processes, being used to refer to a combination of strengths of character encompassing facets of cooperativism (positive personal relationships), self-direction (life purpose, autonomy, environmental domain and self-acceptance) and self-transcendence (personal growth and self-realization) (3, 4). Hedonic is focused on the search for pleasure and happiness, being related to the experience of satisfaction, that is, what makes life pleasurable, the presence of positive affects and the absence of negative affects (3). Despite having different theoretical traditions, the constructs are closely related and influence each other (3).

Thus, instead of focusing in the difference between hedonic and eudaimonic components, these evaluations can be either in terms of affect (positive and negative) or cognitive reflections (5). Positive and negative affects are more related to the frequency with which people experience emotions than with the intensity of those emotions (6). Positive affects relate to pleasing emotions such as enthusiasm, energy, concentration, and satisfaction (7). On the other hand, negative affects are conditions of displeasure that involve mood swings, anger, and distress. The cognitive component of wellbeing is the self-assessment of life experiences, which is developed according to a set of self-imposed standards (7).

Children's Subjective wellbeing has been widely studied the past several decades (8). Some of the studies in children were carried out from the parents' perspective, as if children are unable to properly assess and understand issues related to their own lives (9), or were based on childhood sociology to predict future outcomes (10). However, considering children as present-moment members of the community moving toward future adults, with the right to immediate wellbeing to optimally develop their skills, studies using children-based data are relevant (10).

Childhood is a time of rapid change, and it is at this time that the trajectories of health and wellbeing are established for life, and that will impact adult life (11). Being a stage for the development of psychological difficulties and mental health problems (12, 13). The main challenge for the study of wellbeing in children and adolescents is to seek more sensitive assessment methods according to the stage of development in which the children are, therefore, the identification of characteristics that lead to the beginning, course and result requires projects that involve a younger age group to understand their vulnerabilities (13). Thus, wellbeing and quality of life measuring instruments for children must account for the stage of development of each age (14). An instrument should have different formats that are appropriate for the age group to be assessed, taking into account the normal age limitations and other factors, especially for very young or sick children who may have difficulty in providing accurate information (15). In addition, the response options should be age-appropriate (16). Some instruments use images so that children can more easily identify the best answer (17, 18).

Despite the importance of the knowledge of aspects that influence children's life according to their own point of view and their perception of interpersonal relationships, few instruments are available for measuring these aspects (19). Among them, the *Autoquestionnaire Qualité de Vie Enfant Imagé* (AUQEI) was initially proposed in French by Manificat and Dazord (20) and later translated into Portuguese (17) with the objective of assessing the subjective wellbeing of children between 4 and 12 years of age, based on the premise they are capable of expressing their own feelings. The instrument has been mostly used in children with health problems such as autism (21), cystic fibrosis (22), cerebral palsy (23, 24), spinal muscular atrophy (25), orofacial clefts (26), and born prematurely (27). However, no study has used confirmatory analysis to verify the validity of the data obtained with the AUQEI. The selection of the instrument for subjective wellbeing investigation in children must be guided not only by the pediatric context and the stage of development of the study population (17) but also by adequate estimates of validity and reliability of the data collected with the instrument in the sample to guarantee the quality of the obtained data (28).

The knowledge of this construct is relevant in order to allow for the early identification of children at-risk for poor wellbeing in community or educational settings (29). Therefore, to help support the improvement of child services and development of public policies, this study aimed at evaluating the psychometric properties of the AUQEI when applied to pre-school children and estimate the influence of demographic characteristics on their subjective wellbeing.

## 2. Methods

### 2.1. Ethical aspects

The main investigator of the study obtained agreement of the Children's Education and Recreation Centers (CER) to conduct the study and scheduled the application of the questionnaire to the participating mothers and the interviews with the children. The STROBE tool (Strengthening the Reporting of Observational studies in Epidemiology) was used to assist the study design and data reporting (30).

A signed informed consent form was obtained by all participants. This study was approved by the Research Ethics Committee. To be included in the study, children who agreed to participate had to provide a Consent Form signed by their parents. The study followed the ethical guidelines of the National Health Council Resolution 466/2012.

### 2.2. Study design and sampling

This was an observational, cross-sectional study. Preschool children (4–6 years old) enrolled in municipal public education institutions in Araraquara -São Paulo- Brazil were invited to participate in the study. The sample recruitment was done by the three-stage probabilistic method. In the first stage, the clusters were defined (considering the educational institution), in the second, the sample was stratified according to the number of preschoolers enrolled in the participating centers, and in the third stage, simple probabilistic sampling was performed.

The calculation of the minimum sample size was performed using α = 5%, β = 20%, ε = 10%, *N* = 2.272 (total number of preschool children enrolled in CER), and the prevalence of positive subjective wellbeing of 50%, because a reference value for this parameter was not found in the literature. Thus, the minimum sample size estimated was 329 children, and with a 15% addition to account for a potential 15% loss rate, the final sample size was 388. This sample size was also sufficient to meet the statistical analysis requirements [58 parameters: 26 items, 26 errors, and six correlations between factors; considering the need for 5 subjects per parameter (31), *N* = 290].

### 2.3. Sample characterization

Demographic data (gender, age, education level, marital status, work activity of mothers and economic strata of the family members) were collected by questionnaires answered by the children's mothers. The economic stratum was estimated using the Brazilian Economic Classification Criteria (32), being the participants classified according to their economic strata [mean monthly income: low—C/D/E (U$ 175.63–735.50) and high—A/B (U$ 1,330.09–5,789.67)].

### 2.4. Measuring instrument

**Figure 1:**
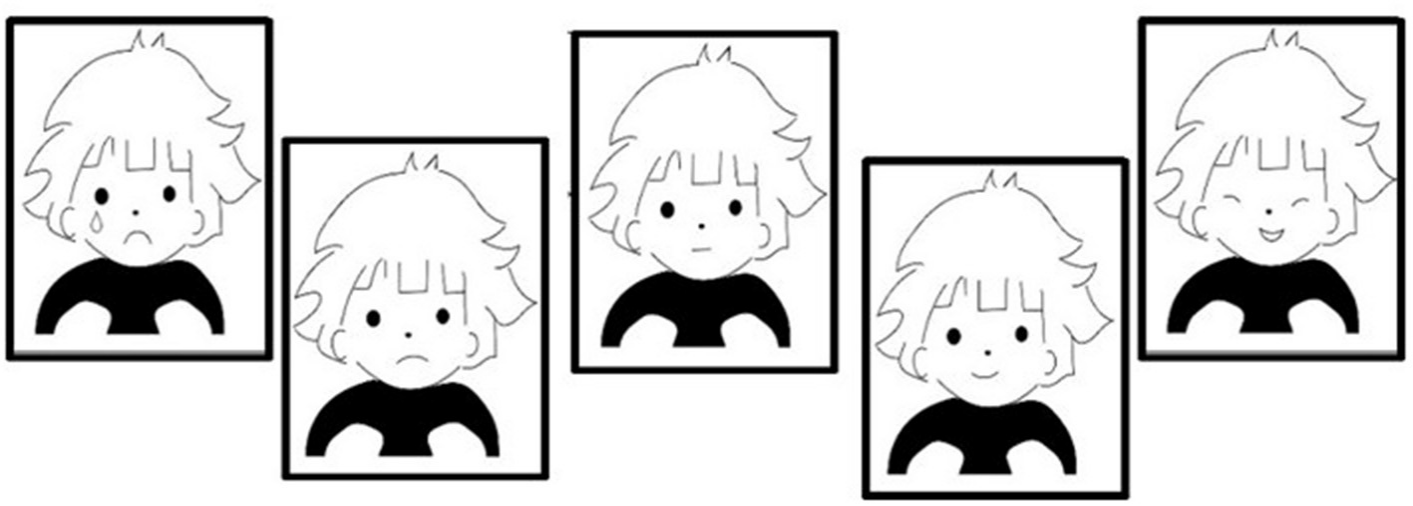
Images for response options of the AUQEI.

The Portuguese version of AUQEI (17) consists of 26 items distributed in four factors (Autonomy, Leisure, Function, and Family). To facilitate the understanding by, and application of, the instrument in children, the responses are represented by images of faces with different emotional states (ranging from very unhappy to very happy). The original version had a response scale with four points without a neutral point. However, in this study, we chose to use a 5-point scale with the inclusion of a neutral point. This choice was based on the fact that previous studies (33–35) indicated that the greater the number of response categories, the better the sensitivity of the items and the greater the probability to discriminate structurally different individuals. In addition, the addition of the neutral point (35) was carried out in order to make respondents more comfortable, as it is possible that for some AUQEI questions the respondent does not have an opinion or experience and, therefore, the answer neutral would be the most viable alternative (Figure 1).

### 2.5. Content validity

The content of the AUQEI items and the response scale were then evaluated by three experts in the areas of pediatrics, psychology and psychometry to estimate the instrument's content validity. The clarity of the items, their practical relevance, theoretical relevance and scope were evaluated, following the proposal of Hernández-Nieto (36). After establishing an absolute consensus among the experts, the AUQEI was applied to the target population in order to verify whether the sentences, instructions and the response scale were understandable for the children and what would be the best format for applying the response scale (Figure 1).

A pilot study was done with 25 children. Individual plastic cards with each of the five faces were presented to children on 3 sizes: 7, 10, and 15 cm long, all 7 cm wide. The cards were arranged sequentially, from the “very unhappy” to the “very happy”. The children were asked about the preferred card size to answer each question, with 88% of children choosing the 10 cm card. During the interview, the researcher asked the children about the difficulties in understanding the content of the items and all the items were well-understood. The children answered each item without time-restriction and indicated the image (answer) that best represented their feeling. The mean duration of the interview with the children was 19.8 (SD = 1.60) minutes.

### 2.6. Evaluation of psychometric indicators

#### 2.6.1. Internal data validity

Data were summarized by means, medians, and standard deviations and assessed for skewness and kurtosis. Strong deviations from normality were considered if skewness and kurtosis were above 3 and 7, respectively (37).

To evaluate the validity and reliability of the data, the total sample was randomly subdivided into two parts, “Test Sample” and “Validation Sample”, and the psychometric properties of the AUQEI were evaluated for the two samples, separately.

The factorial validity was estimated using confirmatory factor analysis (CFA) with the Weighed Least Squares Mean and Variance Adjusted (WLSMV) estimation method. To assess the fit of the model to the data, the chi-square per degrees of freedom ratio (χ^2^/df), the Comparative Fit Index (CFI), the Tucker-Lewis Index (TLI), and the Root Mean Square Error of Approximation (RMSEA) and local adjustment will also be considered based on the assessment of the factor loading of the items (λ) (28, 38, 39) were used. The fit was considered acceptable when χ^2^/df ≤ 5.0, CFI and TLI ≥ 0.90, RMSEA ≤ 0.10 and factor loading (λ) ≥ 0.5 (28, 37, 38). If the model fit was not acceptable, the modification indices calculated using the Lagrange Multiplier (ML) method were observed. Besides the fitness evaluation of the tetra-factorial model (originally proposed for the AUQEI model), the Unidimensional Congruence (UniCo), Explained Common Variance (EVC) and the Mean of Item Residual Absolute Loadings (MIREAL) were assessed to verify the overall fit of the unidimensional model to the data. Values of UniCo > 0.95, EVC > 0.85, and MIREAL <0.30 were considered to indicate an adequate fit of the unifactorial model to the data (40). The replicability of the model to future studies was estimated using the H-index (H-latent: assesses how well the factor can be identified by the continuous latent response variables that underlie the observed item scores; H-observed: assesses how well it can be identified from the observed item scores) (41). Values >0.80 indicate that the items adequately represent the factor and the structure has a high probability of being replicated (41).

Convergent validity was assessed based on the average variance extracted (AVE). AVE was estimated using the proposal by Fornell and Larcker (42) and was considered adequate if ≥0.50.

The MPLUS v.8.3 (Muthén and Muthén, Los Angeles, CA) and FACTOR (Lorenzo-Seva and Ferrando, Tarragona, Spain) programs were used to perform the analyses.

AUQEI's reliability was estimated from the ordinal alpha (α) and omega (ω) coefficients calculated with the “semTools” package (43) and “lavaan” (44) in the R program (45). The values α and ω ≥ 0.70 were indicators of acceptable internal consistency (31).

To assess whether the factors obtained were constant in independent samples, factorial invariance was carried out through multi-group analysis. The model for the “Test Sample” was compared with the model of the “Validation Sample”. The CFI difference (ΔCFI) was used to compare factor loading (λ), thresholds (*t*), and variance/covariance of the residuals (Cov/Res). The CFI values of the configural models (M0), the factor loading model (M1), the threshold model (M2), and the residual model (M3) were considered (Metric invariance: M1 − M0; Scalar invariance: M2 − M1; and Strict invariance: M3 − M2) (46). Invariance between the models was confirmed when the CFI difference (ΔCFI) was <0.01 (47).

#### 2.6.2. Validity in relation to external variables

The *Satisfaction with Life Scale* (SWLS) was used to assess the validity of the AUQEI model in relation to external variables, based on the responses of the children's mothers taking part in the study and on the children's ceo-d index. The SWLS was originally developed in English (48), characterized as a unifactorial scale consisting of five items according to a 7-points Likert scale (1: fully disagree to 7: fully agree). Both the AUQEI model and the SWLS should ideally be positively correlated, with the better the mother's wellbeing the greater the child's wellbeing (positive convergent validity). Negative convergent validity was estimated between the AUQEI model and the ceo-d index (decayed, indicated extraction or filled—ceo-d index) (49). It is anticipated that the AUQEI model and a health measure the ceo-d index present a negative and significant correlation, where the higher the ceo-d index the lower the child's wellbeing. The use of the SWLS and the ceo-d index to estimate the validity of the model was chosen out of convenience, as the present study is part of a wider project for which these variables had been collected. In the wider study, the SWLS was used to assess the subjective wellbeing of the children's mothers taking part in the present study, while the ceo-d index was used to measure the experience of caries.

#### 2.6.3. Structural model and subjective wellbeing of preschool children

A structural model was made considering the impact of demographic variables on the AUQEI. The variables sex of the child (male or female), age of the mother, marital status (married or not married) and family economic strata [low - C/D/E (mean monthly income: U$ 175.63–735.50) and high - A/B (U$ 1,330.09–5,789.67] were included in the model.

The goodness of fit of this hypothetical model was evaluated on the polychoric correlation matrix using Weighed Least Squares Mean and Variance Adjusted (WLSMV) estimation, as implemented in the R program (45). The fitting of the structural model was evaluated using the previously cited indexes (χ^2^/df, CFI, TLI, and RMSEA) (28). The trajectories (β) were estimated and evaluated with the *z*-test. A significance level of 5% (28).

In addition, the average scores for the general sample were calculated and an average subjective wellbeing ≥2.46 was considered positive subjective wellbeing, based on the suggestion of the instrument's author of using the 61.5 percentile (P61.5) as a reference for adequate subjective wellbeing. Based on this recommendation, the prevalence of positive subjective wellbeing (adequate) was estimated using a 95% confidence interval (95% CI). The authors of the scale recommend the assessment of scores using the sum. However, as psychometric instruments need to be fitted to the data and context under assessment and there was a risk of “losing items”, we did not use the sum of items as score but the average, despite using the cutoff percentile suggested by the authors. The average scores were also calculated for each item of the instrument by a 95% confidence interval (95%CI). The level of significance was 5%.

## 3. Results

A total of 443 children participated in the study [mean age: 5.19 Standard deviation (SD = 0.64) years; 52.4% male]. The average age of the participants' mothers was 33.4 (SD = 7.01) years and most were married, had a job, and belonged to economic strata B and C. The demographic information of the sample is shown in Table 1.

**Table 1:** Demographic characteristics of the study participants.

**Characteristic**	***n* (%)**	**Test (*n* = 212)**	**Validation (*n* = 231)**
**Children**			
**Sex**			
Male	232 (52.4)	107 (50.5)	125 (54.1)
Female	211 (47.6)	105 (49.5)	106 (45.9)
**Mothers**			
**Age (years)**			
<30	163 (37.9)	85 (41.3)	78 (34.8)
≥30	267 (62.1)	121 (58.7)	146 (65.2)
**Marital status**			
Single	124 (28.6)	63 (30.4)	61 (26.9)
Married	276 (63.6)	129 (62.3)	147 (64.8)
Divorced	30 (6.9)	12 (5.8)	18 (7.9)
Widow	4 (0.9)	3 (1.4)	1 (4.0)
**Work activity**			
No	142 (32.4)	65 (31.3)	77 (33.5)
Yes	296 (67.6)	143 (68.8)	153 (66.5)
**Economic strata (estimated mean family income)**			
A (U$ 5,789.67)	23 (5.2)	8 (3.8)	15 (6.5)
B (U$ 1,330.09–2,575.89)	200 (45.1)	89 (42.0)	90 (39.0)
C (U$ 419.54–735.50)	205 (46.3)	110 (51.9)	116 (50.2)
D-E (U$ 175.63)	15 (3.4)	5 (1.4)	10 (4.3)

The descriptive statistics of the answers given to the AUQEI items by the participants of the two samples (Test and Validation) are shown in Table 2. As none of the AUQEI items presented absolute values of skewness > 3 and kurtosis > 7, no strong deviation from normality was assumed, and therefore the psychometric sensitivity of the items was considered adequate.

**Table 2:** Descriptive statistics of the Autoquestionnaire Qualité de Vie Enfant Imagé (AUQEI) responses by the participants (test sample *N* = 212 and validation sample *N* = 231).

	**Test sample/validation**					
**Item**	**Mean**	**Standard deviation**	**Skewness**	**Kurtosis**	**Minimum**	**Maximum**
**Say how you feel:**						
It1. at the table, with your family	2.99/3.05	0.98/0.94	−0.58/−0.68	−0.74/−0.47	1/1	4/4
It2. at night when you lie down	2.42/2.44	1.00/1.01	−0.59/−0.20	−1.00/−0.84	0/0	4/4
It3. if you have siblings, when you play with them	3.12/3.15	0.83/0.86	−0.64/−0.78	−0.20/0.29	0/0	4/4
It4. at night, when sleeping	2.39/2.45	1.03/0.99	−0.04/−0.18	−1.00/−0.86	0/0	4/4
It5. in the classroom	2.84/2.86	0.88/0.85	−0.36/−0.20	−0.58/−0.78	1/1	4/4
It6. when you see a picture of yourself	2.97/3.00	0.80/0.80	−0.21/−0.19	−0.80/−0.95	1/1	4/4
It7. at play time, during school recess	3.51/3.48	0.56/0.58	−0.76/−0.71	−0.50/0.30	1/1	4/4
It8. when you go to a doctor's appointment	1.83/1.90	1.27/1.16	−0.04/−0.05	−1.22/−1.09	0/0	4/4
It9. when you play a sport	3.27/3.32	0.78/0.77	−0.89/−0.84	0.30/−0.10	1/1	4/4
It10. when you think of your father	2.96/3.08	1.07/0.97	−0.81/−0.72	−0.22/−0.45	0/0	4/4
It11. on your birthday	3.47/3.46	0.67/0.67	−1.26/−0.95	1.87/0.07	1/1	4/4
It12. when you do your homework	2.85/2.94	0.95/0.93	−0.47/−0.42	−0.22/−0.66	0/0	4/4
It13. when you think of your mother	3.31/3.29	0.76/0.79	−1.31/−1.06	2.82/1.41	0/0	4/4
It14. when you are admitted to the hospital	1.02/1.10	0.89/0.92	0.27/0.54	−0.90/−0.32	0/0	4/4
It15. when you play alone	2.18/3.16	1.23/1.18	−0.41/0.12	−1.35/−1.35	0/0	4/4
It16. when your dad or mom talk about you	2.72/3.72	1.20/1.14	−0.57/−0.64	−0.74/−0.45	0/0	4/4
It17. during sleepovers	2.46/3.46	1.24/1.25	−0.49/−0.52	−0.87/−0.86	0/0	4/4
It18. when someone asks you to show something you know how to do	3.07/4.03	0.86/0.87	−0.80/−0.60	0.76/0.01	0/0	4/4
It19. when friends talk about you	2.74/3.73	1.12/1.10	−0.66/−0.72	−0.53/−0.40	0/0	4/4
It20. when you take medicines	1.64/2.59	1.02/1.10	0.26/0.24	−0.37/−0.56	0/0	4/4
It21. during the holidays	3.41/4.36	0.78/0.77	−1.11/−1.10	0.36/0.60	1/1	4/4
It22. when you think of being a grown-up	3.11/4.13	0.82/0.83	−0.57/−0.58	−0.08/−0.24	0/0	4/4
It23. when you are away from your family	2.02/2.95	1.11/1.10	0.30/0.39	−0.76/−0.66	0/0	4/4
It24. when you get grades from school	3.11/4.10	0.83/0.89	−0.35/−0.56	−1.09/−0.70	1/1	4/4
It25. when you are with your grandparents	3.42/4.44	0.67/0.69	−0.80/−1.07	−0.30/0.77	1/1	4/4
It26. when you watch television	3.54/4.53	0.58/0.62	−0.83/−0.99	−0.29/−0.06	2/2	4/4

**Figure 2:**
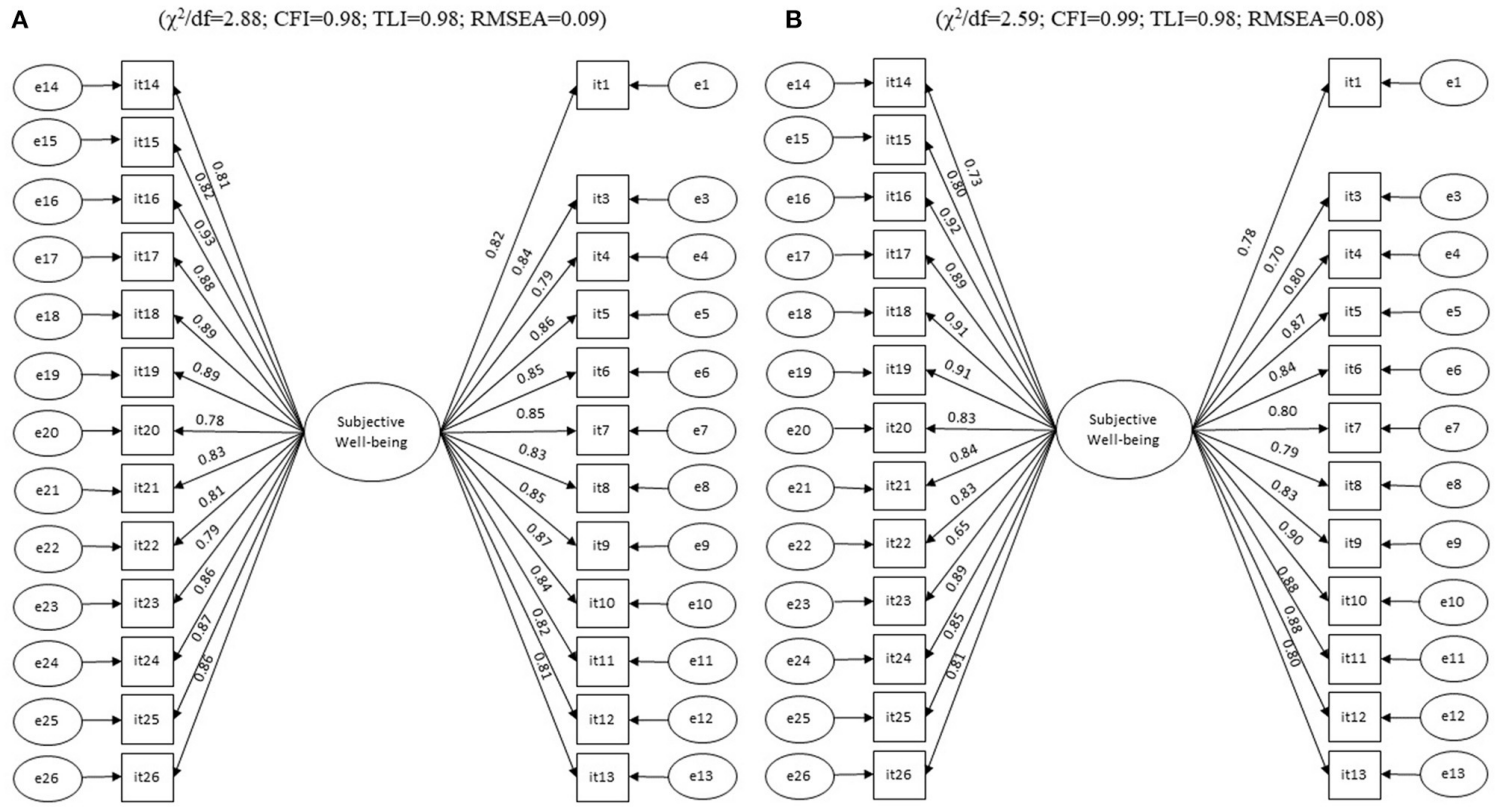
Factorial model fitted for the test sample (*n* = 212) **(A)** and validation sample (*n* = 231) **(B)** of preschool children.

Despite the adequate fit of the original AUQEI model (complete - M) to the total sample, the factors Leisure and Autonomy [Variance Inflation Factor (VIF) = 166.91], Function and Autonomy (VIF = 12.75), Function and Leisure (VIF = 125.25), Family and Autonomy (VIF = 55.80), Family and Leisure (VIF = 38.21), and Family and Function (VIF = 100.25) were collinear. In addition, a high correlation was found between the errors of items 2 and 3 of the instrument, which led to the non-convergence of the covariance matrix. Thus, each item (2 and 3) was excluded individually and then both were excluded, however, this strategy did not favor the matrix's convergence. Collinearity may suggest that the AUQEI applied to this study sample has a single factor model and the adequacy verification indices of the one-dimensional model reinforce this suggestion (UniCo = 0.995; ECV = 0.946; MIREAL = 0.161), therefore, this proposal was tested. In the single factor model, was observed high correlation between items 2 and 3, we excluded item 2, based on the modification indices and the theoretical content of the item. The single-factor model showed adequate fit to the sample as well as convergent validity and adequate reliability both in the total sample (λ = 0.71–0.92; χ^2^/df = 4.47; CFI = 0.98; TLI = 0.98; RMSEA = 0.08; AVE = 0.70; α = 0.98; ω = 0.93) and in the Test and Validation samples (Figure 2). This model has a high probability of being applied in other studies (H-Latent = 0.984; H-Observed = 0.946).

In the analysis performed on independent samples (Test, *n* = 212 × Validation, *n* = 231), strict invariance was observed (ΔCFI_M1 − M0_ = 0.001; ΔCFI_M2 − M1_ = 0.000; ΔCFI_M3 − M2_ = 0.001) indicating that the factorial structure found remains in independent samples. The structural model tested presented adequate fit to the sample (χ^2^/df = 3.58; CFI = 0.98; TLI = 0.99; RMSEA = 0.07). The child's sex, the mother's age, the family's economic level, the fact that the mother is or is not married, and the exercise of work activity did not impact the children's wellbeing (Table 3).

**Table 3:** Structural model for assessing demographic contribution to children's subjective wellbeing (AUQEI).

**Pathway**	**β**	**β_S_**	**SE**	***p***
Sex → AUQEI	−0.019	−0.010	0.104	0.520
Age → AUQEI	0.002	−0.013	0.008	0.803
Work activity → AUQEI	0.201	0.092	0.116	0.082
Marital status → AUQEI	−0.115	−0.054	0.111	0.300
Economic strata → AUQEI	0.113	0.056	0.108	0.297

βS, β standardized; SE, standard error.

The correlational analysis between the AUQEI and the SWLS (*r* = 0.70, *p* < 0.001) pointed to an adequate positive convergent validity of the AUQEI, while the correlation between the AUQEI and the ceo-d index (*r* = −0.36, *p* < 0.001) to attest negative convergent validity of the AUQEI model.

**Figure 3:**
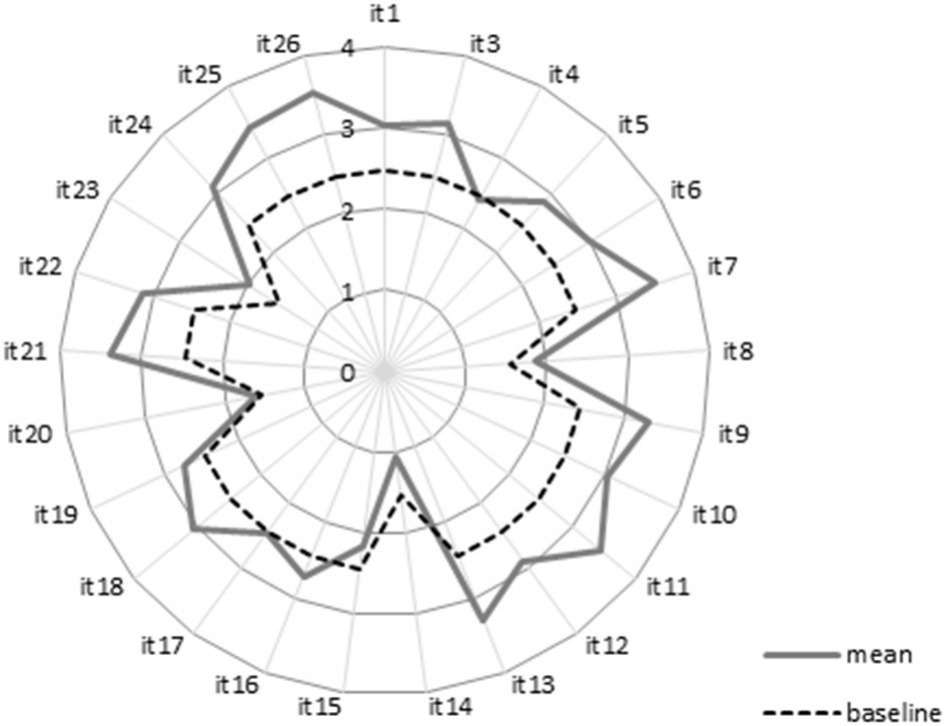
Mean item score for the AUQEI given by children.

Most children [76.7%; (95% CI: 72.7–80.5%)] had positive subjective wellbeing. The mean scores of the items from children (*n* = 443) and the baseline (P61.5; mean score = 2.46) are shown in Figure 3.

Higher scores of subjective wellbeing were given for items concerning recreation, vacations, and birthdays, and lower scores referred to hospitalization, medication, medical consultation, and being away from the family. This corroborates the theoretical framework of the instrument, pointing toward adequate levels of subjective wellbeing. Only item 14 (hospitalization) presented a lower score than the baseline.

## 4. Discussion

The present study confirmed the validity and reliability of subjective wellbeing data of preschool children obtained with the AUQEI. The found wellbeing estimates highlight the feasibility and importance of assessing subjective wellbeing of young children and expand the knowledge required for developing educational programs for this population.

In recent years, the study of children subjective wellbeing has increased (17, 20, 23), however, it is still a challenge to choose the best measurement tool to assess the wellbeing, especially in young children. Although the AUQEI is an interesting instrument to assess wellbeing in children (17, 20), there were still no studies that supported the validity and reliability of the measure obtained with this instrument. Some important aspects in the validation process, such as item analysis, psychometric sensitivity, and factorial validity, were not performed, which may compromise the conclusions drawn from previous studies.

The AUQEI factorial model that fitted the data adequately was the single factor structure. To obtain the best fit, a new theoretical proposal different from the original one was used. The validation and reliability analysis of the original (four-factor) and the single-factor versions were conducted, and a high correlation among the factors of the original structure was found, which compromises the variances of the parameter estimates. When at least one of the variables is redundant, an estimate of negative variance occurs (28, 37). Therefore, to avoid problems in model estimation, we tested the single-factor model, which adequately fitted the data. This proposal also provided adequate validity of the AUQEI in relation to external variables, ensuring, therefore, three measures of validity to the instrument (content, internal structure and external variables). This finding should raise the alert for future users of the instrument about the importance to obtain estimates of adequate quality for each sample and study context. The process of adapting an instrument for a population is only completed after confirmation of validity and reliability applied to different samples (28, 37). The comparison of the present findings with those of others was not possible since no previous study verified the fit of the AUQEI factor model to other samples using a confirmatory analysis.

Item 2 (“Say how you feel: at night, when you lie down”) presented a high correlation with item 3 (“Say how you feel: if you have siblings, when playing with them”), and we found that item 2 had a high theoretical similarity to item 4 (“Say how you feel: at night, when sleeping”) causing collinearity, which may have resulted in the high correlation between items 2 and 3. Considering that such young children, in our sample, could not differentiate the concepts in items 2 and 4, item 2 was excluded. Evidence from the literature on child development and psychology shows that children under 6 years of age have a limited distinction of language and verbalization of emotions, especially when referring to past events, and abstract thinking capacity develops after the age of 6 (50, 51). Future studies that include children from different stages of development are suggested to assess the best theoretical/factorial proposal for the AUQEI in each of these phases.

The strong invariance of the AUQEI observed between independent samples confirmed the stability of the single-factor model applied to young children. Thus, a valid and reliable set of data obtained with the AUQEI model is provided.

Currently, investigation of wellbeing has shifted, aiming at individuals without a specific complaint or disease, focusing on promotion of wellbeing and not just its impair (52). However, most of the studies are carried out on children with health problems (20, 23, 24). So far, only the study by Assumpção et al. (17) was performed in healthy children; however, the authors did not present evidence related to the validity of the proposed model. In addition, their model was developed for a sample of children at different stages of development (4–12 years old), which may explain the difference between the data obtained in the present study in relation to the factorial model. In the study by Assumpção et al. (17) only 49 children were aged 4–6 years and, therefore, their data may have a low contribution in the 4-factor model presented. Thus, the present study contributes to the field of study by providing a specific model for children under 6 years of age; further studies may confirm or contest our findings. In addition, future studies aiming at defining the best model for assessing subjective wellbeing using the AUQEI in children at different stages of development are recommended.

No difference in the scores of subjective wellbeing was found between mothers' age, marital status, work activity, and income (see structural model Table 3). Although income has a role in wellbeing, it is not its main indicator (19), and other conditions such as culture and social aspects should be considered in the relationship between income and wellbeing (53). The lack of an effect of the above variables in the responses shows that children do not have a clear perception of the interference of these conditions in their daily life, and provide a more self-focused assessment, based on the effects of their own world on the daily routine, without considering broader aspects.

Children presented a greater wellbeing score for items related to recreation, vacations, and birthdays, a finding that corroborates with previous studies (17, 54). Activities that break the routine and provide fun moments, such as free-time activities, are valued in childhood, being significant for wellbeing, especially among younger children (19). In contrast, the items with the lowest scores were those that refer to hospitalization and being away from the family, which is in line with the work of Assumpção et al. (17) not only in relation to the items, but also with regard to the overall positive wellbeing for most children. Despite presenting lower scores, the average subjective wellbeing was positive for most participants, which is explained by the concept of subjective wellbeing being a balance between positive and negative emotions, which not always is affected by some conditions, be they material, health-related, financial, or security-related. The influence of these factors depends on expectations and values of each person, the people around him or her, and the community in which he or she lives. Therefore, subjective wellbeing involves a global judgment of all aspects of life, and although some conditions can affect wellbeing, the emphasis is usually placed on the general judgment of a person's life (55). Despite the high prevalence rate of subjective wellbeing found in the sample, approximately one-fourth of the sample presented lower values than expected. Thus, further monitoring and investigation of the factors involved is suggested.

As limitation of the study, the data were collected in a single national context, namely, Brazil. To broaden the scope of research, it is recommended that further studies be conducted using samples from different national contexts. While the main factorial structure is expected to be consistent, cross-cultural comparisons may unveil variations in factor loadings and associations with other variables, fostering discussions on sociocultural factors' role in children's wellbeing. In addition, the cross-sectional design of the study does not allow the establishment of a cause-and-effect relationship. Despite this limitation, this study provides information about the psychometric properties of the AUQEI model for pre-school children and identifies the subjective wellbeing of preschool children, which can contribute to health professionals and researchers from different areas of knowledge in guiding and developing preventive strategies focusing not only on treating health problems, but also in promoting wellbeing.

The AUQEI model provided valid and reliable data, being thus an interesting instrument to assess the wellbeing of young children. This assessment can broaden the view of education and health professionals in order to better estimate, re-establish and monitor the wellbeing of children. The demographic characteristics of the mother or family analyzed in the present study, or even the child's sex, had no significant impact on the children's wellbeing. The study observed a high prevalence of children reporting a high subjective wellbeing.

## Data availability statement

The raw data supporting the conclusions of this article will be made available by the authors, without undue reservation.

## Ethics statement

The studies involving human participants were reviewed and approved by Research Ethics Committee of São Paulo State University (UNESP). Written informed consent to participate in this study was provided by the participants' legal guardian/next of kin.

## Author contributions

BS and BM involved in conception and design, data acquisition, analysis and interpretation, and drafted and critically revised the manuscript. JM, JC, and LC involved in conception and design, data interpretation, and drafted the manuscript. All authors contributed to the article and approved the submitted version.
